# Systemic FasL and TRAIL Neutralisation Reduce Leishmaniasis Induced Skin Ulceration

**DOI:** 10.1371/journal.pntd.0000844

**Published:** 2010-10-12

**Authors:** Geremew Tasew, Susanne Nylén, Thorsten Lieke, Befekadu Lemu, Hailu Meless, Nicolas Ruffin, Dawit Wolday, Abraham Asseffa, Hideo Yagita, Sven Britton, Hannah Akuffo, Francesca Chiodi, Liv Eidsmo

**Affiliations:** 1 Ethiopian Health and Nutrition Research Institute (EHNRI), Parasitology Laboratory, Addis Ababa, Ethiopia; 2 Department of Microbiology, Tumour and Cell Biology, Karolinska Institutet, Stockholm, Sweden; 3 Transplantationslabor, Klinik für Viszeral- und Transplantationschirurgie, Medizinische Hochschule Hannover, Hannover, Germany; 4 St. Paulos General Specialized Hospital, Addis Ababa, Ethiopia; 5 Armauer Hansen Research Institute (AHRI), Addis Ababa, Ethiopia; 6 Department of Immunology, Juntendo University School of Medicine, Tokyo, Japan; 7 Unit of Infectious Diseases, Department of Medicine Solna, Karolinska Institutet, Stockholm, Sweden; 8 Unit of Dermatology and Venerology, Department of Medicine Solna, Karolinska Institutet, Stockholm, Sweden; Institut Pasteur, France

## Abstract

Cutaneous leishmaniasis (CL) is caused by *Leishmania* infection of dermal macrophages and is associated with chronic inflammation of the skin. *L. aethiopica* infection displays two clinical manifestations, firstly ulcerative disease, correlated to a relatively low parasite load in the skin, and secondly non-ulcerative disease in which massive parasite infiltration of the dermis occurs in the absence of ulceration of epidermis. Skin ulceration is linked to a vigorous local inflammatory response within the skin towards infected macrophages. Fas ligand (FasL) and Tumor necrosis factor-related apoptosis-inducing ligand (TRAIL) expressing cells are present in dermis in ulcerative CL and both death ligands cause apoptosis of keratinocytes in the context of *Leishmania* infection. In the present report we show a differential expression of FasL and TRAIL in ulcerative and non-ulcerative disease caused by *L. aethiopica*. *In vitro* experiments confirmed direct FasL- and TRAIL-induced killing of human keratinocytes in the context of *Leishmania*-induced inflammatory microenvironment. Systemic neutralisation of FasL and TRAIL reduced ulceration in a model of murine *Leishmania* infection with no effect on parasitic loads or dissemination. Interestingly, FasL neutralisation reduced neutrophil infiltration into the skin during established infection, suggesting an additional proinflammatory role of FasL in addition to direct keratinocyte killing in the context of parasite-induced skin inflammation. FasL signalling resulting in recruitment of activated neutrophils into dermis may lead to destruction of the basal membrane and thus allow direct FasL mediated killing of exposed keratinocytes *in vivo*. Based on our results we suggest that therapeutic inhibition of FasL and TRAIL could limit skin pathology during CL.

## Author Summary

Cutaneous leishmaniases are associated with parasite-induced inflammatory lesions of the skin. The degree of clinical pathology is not associated with parasitic burden; on the contrary, ulcerative lesions are associated with low infectious load, and non-ulcerative lesions are associated with an abundant parasite infiltration. *Leishmania* are intracellular parasites in mammalian hosts and reside in macrophages in the deep layers of the skin, the dermis. The exact mechanism of ulceration in CL is not known and *Leishmania* parasites do not directly induce destruction of keratinocytes in the most superficial layer of the skin, the epidermis. In this study we investigated if ulcerated lesions were associated with higher expression of FasL- and TRAIL-induced cell-death of keratinocytes. We found a higher expression of FasL and TRAIL in human skin samples from ulcerative as compared to non-ulcerative leishmaniasis. In a mouse model of ulcerative leishmaniasis neutralisation of FasL and TRAIL reduced ulceration. We suggest that FasL and TRAIL participate in the ulcer formation during leishmaniasis both as a chemoattractant of activated neutrophils leading to tissue destruction and through direct killing of keratinocytes. Possible approaches to use this concept in therapeutical interventions with the aim to reduce immunopathology associated with leishmaniasis are discussed.

## Introduction

Leishmaniasis is a group of parasitic diseases associated with heterogeneous clinical manifestations. Symptoms range from lethal disease with overwhelming infection of the bone-marrow, spleen and liver to localised self-healing ulcers of the skin. *Leishmania aethiopica* is the main causative agent of CL in the highlands of Ethiopia. Upon infection, parasites reside and replicate within tissue macrophages during an initial silent phase of the infection and the clinical presentation of CL is mainly associated with the infiltration of circulating inflammatory cells into infected tissues. *L. aethiopica* infection leads to localised cutaneous leishmaniasis (LCL) or diffuse cutaneous leishmaniasis (DCL). LCL is characterised by erosive ulcers and a strong T cell mediated response [1] which typically results in spontaneous healing within a year, scar formation and solid protection against re-infection [2]. In contrast, DCL is linked to non-ulcerative chronic nodular disease with abundant parasitic infiltration of the dermal compartment of the skin and antigen specific T cell unresponsiveness [3], [4]. Structural differences [5] as well as different immunogenic properties [4], [6] between LCL and DCL causing parasites have been reported. The mechanisms of tissue destruction during ulcerative cutaneous leishmaniasis have not been fully clarified. We have previously reported that dermal FasL and TRAIL expressing cells are present in ulcerative *L. major* infection and that the number of FasL expressing dermal cells correlate to the level of epidermal apoptosis. Furthermore, i*n vitro* experiments propose FasL and TRAIL as major players inducing apoptosis in keratinocytes during *Leishmania* induced inflammation [7], [8].

In the present study expression of FasL and TRAIL within the skin was investigated in ulcerative and non-ulcerative manifestations of *L. aethiopica* induced CL. More FasL and TRAIL expressing cells were detected in ulcerative self-healing LCL as compared to non-ulcerative chronic DCL. In line with these results, neutralisation of FasL and TRAIL *in vivo* during experimental leishmaniasis in BALB/c mice led to reduction of ulceration and was not associated with increased infective loads or increased spread of the infection through the lymphatics.

## Materials and Methods

### Ethical statement

This study was conducted according to the principles expressed in the Declaration of Helsinki. The study was approved by the Institutional Ethical Review Board of Karolinska Institutet (reference number 31-5427/08) and by The National Ethical Clearance Committtee (NECC) at the Ethiopia Science and Technology Commission (reference number: RDHE/78-43/2002). All patients provided written informed consent for the collection of samples and subsequent analysis. All animals were handled in strict accordance with good animal practice as defined by the relevant national animal welfare bodies, and this study was approved by the Regional Animal Studies Ethical Committee, Stockholm North, Sweden (reference number N72/05 and 305/08).

### Patient material

Skin biopsies were collected from healthy controls at St.Paulos General, Specialized Hospital, Addis Ababa, Ethiopia, and from Ethiopian CL patients at the Armauer Hansen Research Institute (AHRI), Addis Ababa, Ethiopia.

### Immunohistochemistry of skin biopsies

FasL and TRAIL were visualised in formalin fixed tissue as previously described [7], [8] and evaluated in Leica fluorescent microscope. Photomicrographs were obtained using a Zeiss Axioskope 2, AxioVision 4.6 (Zeiss) and processed using Photoshop CS4. Apoptosis was assessed through visualizing fragmented DNA using TUNEL (TdT-mediated dUTP nick end labeling) kit according to the manufacturer's instructions (Roche, Penzberg, Germany). The number of FasL expressing cells was counted in 25× objective and apoptotic cells were counted in 40× objectives, with more than ten fields evaluated per sample. A scoring system for the wide-spread TRAIL expression found was used as shown in Figure S1 and all samples were evaluated blindly.

### Induction of keratinocyte apoptosis *in vitro*

*Leishmania* promastigotes propagated from ulcerative and non-ulcerative lesions were used to stimulate healthy peripheral blood mononuclear cells (PBMC) for 7 days at 1∶1 ratio and supernatants were collected and cryopreserved. Supernatants were added to cultures of the keratinocyte cell-line HaCaT [9] for 20 hrs and early apoptotic cells were assessed by AnnexinV/Propidium Iodide staining by microscopy. Fas-activating monoclonal antibody (1 µg/ml, CH-11; MBL, Nagoya, Japan) and recombinant TRAIL (250 ng/ml, R&D Systems) were used as positive controls. Fas-blocking monoclonal antibody ZB4 (1-2 µg/ml, MBL) and TRAIL-blocking antibody 2E5 (2.5 µg/ml, Alexis, KeLab, Gothenburg, Sweden) were added 30 minutes prior to supernatants. Isotype control antibodies to CH-11, ZB4 or 2E5 did not affect keratinocyte apoptosis. Apoptosis was assessed by counting 10 to 20 fields under ×40 ocular magnification and expressed as the number of apoptotic cells per 10 fields.

### *In vivo* model of murine ulcerative leishmaniasis

Infective-stage metacyclic promastigotes of *L. major* (strain Friedlin V1 or LV39, both gift from David Sacks, NIAID, NIH, Bethesda, USA) were isolated from stationary cultures (4–5 days old) by negative selection using peanut agglutinin (Vector Laboratories, Burlingame, CA, USA) as previously described [10].

### Mice

Female BALB/c aged 6–8 weeks were infected intradermally with 5×10^4^ metacyclic *L. major* [11]. Neutralising hamster anti-mouse FasL (MFL-4) [12], rat anti-mouse TRAIL (N2B2) [13] or isotype-matched hamster or rat IgG control (Rockland) were injected *i.p.* at a dose of 0.5 mg twice per week for 4–5 weeks after infection. The evolution of the lesion was monitored weekly by measuring the diameter of the indurations of the ear lesion with a direct-reading vernier caliper (Thomas Scientific, Swedesboro, NJ, USA). After euthanization both ears and retromaxillar lymph nodes were removed. Groups of five mice were infected at three (MFL-4 and isotype-control) and two (N2B2 and isotype-control) different times.

### Estimation of parasite load in ear and retromaxillar lymph nodes

Parasite titrations were performed as previously described [11]. The number of viable parasites in each sample was defined as the highest dilution at which promastigotes could be grown out after 7 days of incubation at 26°C.

### Composition of cellular infiltrate and IFN-γ production in ear and lymph node

Single cell suspension of ear and lymph node tissue was prepared as previously described [11]. Cells were stained ex-vivo for Ly6C-PerCP, CD11b-APC, CD11c-FITC, FasL-PE, (all from BD Biosciences) and CD45-eFluor 450 (eBioscience) Dead cells were excluded by YFD conjugated Live/Dead stain kit (Molecular Probes).

To assess IFNγ producing cells, single cell suspension from ear and lymph nodes were cultured over night (18 hours) in the presence or absence of antigen pulsed dendritic cells as previously described [11]. GolgiStop (BD Biosciences) was added during the last 4 hours of culture. Cells were stained for TCRβ-FICT, CD8-PerCP, IFNγ-APC (BD Biosciences) CD45- eFluor 450, CD4-PECy7 (eBioscience) and Live/Dead (Molecular Probes). 20% (ex vivo staining) and 30% (cytokine restimulation) of the total number of cells per ear and 500 000 events from lymph nodes were acquired on CyAn (Beckman Coulter) and analyzed by FlowJo 8 (Tree Star Inc).

Statistical analysis was performed using Prism Graph Pad Software (Inc. Oberlin Drive, San Diego, USA).

## Results and Discussion

### Clinical presentation of ulcerative and non-ulcerative CL

**Figure 1:**
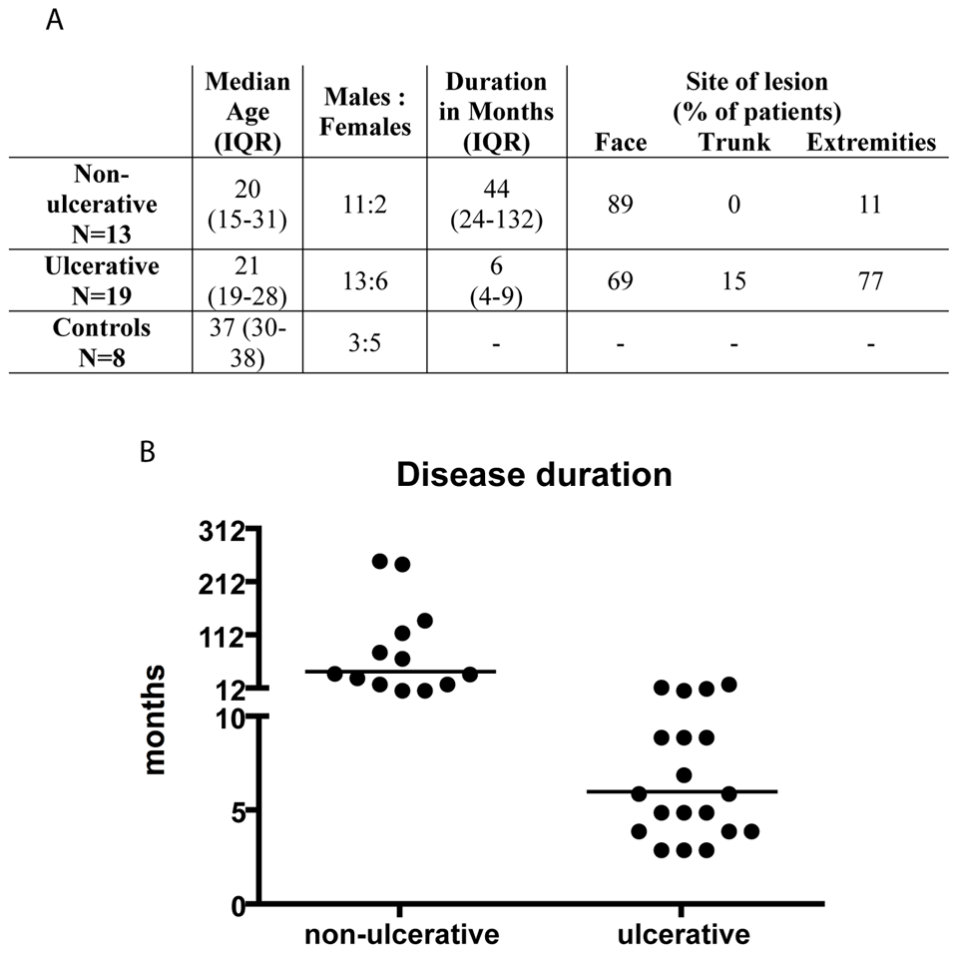
Clinical presentation of non-ulcerative and ulcerative CL. (A) Clinical characteristics of non-ulcerative and ulcerative CL at the time of sample collection. Median and the interquartile range (IQR) are shown. (B) Dot-plot diagram depicting disease duration of non-ulcerative and ulcerative CL at the time of biopsy. The horizontal bar represents the median disease duration.

**Figure 2:**
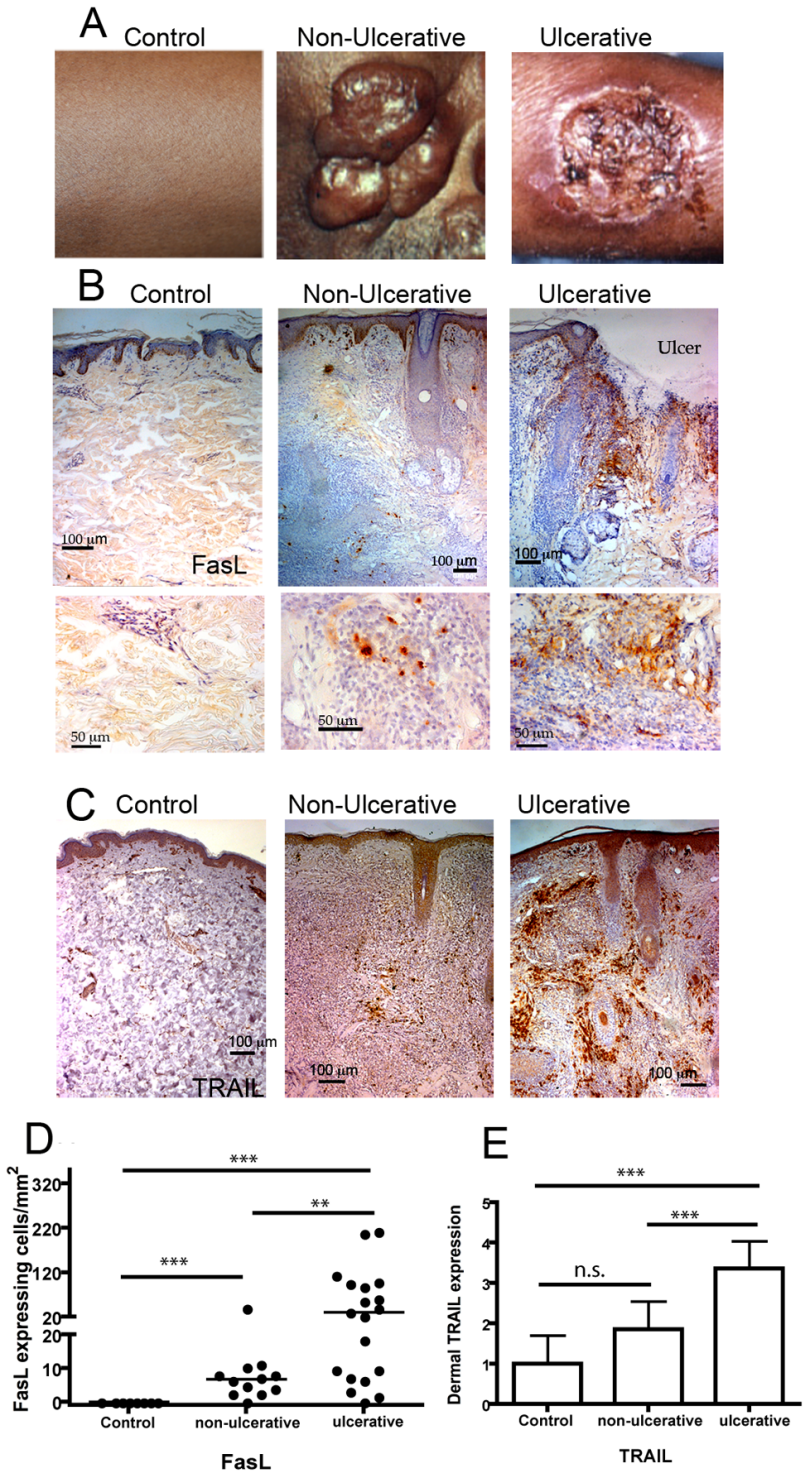
FasL and TRAIL expressing cells infiltrate dermis of ulcerative CL. (A) Photographs of representative non-ulcerative (center) and ulcerative (right) lesions of CL caused by *L. aethiopica*. Control skin (left) was obtained from a fore arm, non-ulcerative from a facial lesion and the ulcerative lesion from a fore arm. (B) FasL expressing cells (DAB, brown) in healthy and lesional skin depicted and counterstained with hematoxilin (blue). The upper row was taken using a 10× objective and scale bars represent 100 micrometers. The lower row show high magnification (40× objective) of FasL expressing cells in dermis. The ulcerated area is marked. (C) TRAIL expressing cells (DAB, brown) counterstained with hematoxilin (blue). (D) Enumeration of dermal FasL+ cells by counting of 10–20 fields per sample at 25× magnification. (E) Dermal TRAIL expression was assessed using an arbitrary scale where 0 represented no signal and 5 represented maximal signal. ***p<0.001, **p<0.01, *p<0.05, ns = not significant.

Skin biopsies from ulcerative (n = 19), non-ulcerative (n = 13) and healthy controls (n = 8) were collected at Armauer Hansen Research Institute and at St. Paulos General Specialized Hospital, both Addis Ababa, Ethiopia. Lesions were designated as ulcerative or non-ulcerative according to clinical presentation (Fig. 1A and 2A). The duration of ulcerative and non-ulcerative lesions was partly overlapping with a median clinical history of lesion formation of 6 vs. 44 months at the time of biopsy (Fig. 1B). All included individuals displaying the non-ulcerative phenotype had lesions in several distinct parts of the body whereas ulcerative disease was confined to a single lesion predominately on the face (Fig. 1A). *Leishmania* infection was verified by detection of amastigotes by May-Grünwald-Giemsa staining or by detection of viable promastigotes in cultures of lesion scrapings. Non-ulcerative lesions contained numerous disorganised macrophages laden with *Leishmania* amastigotes, few lymphocytes and marked plasma cell infiltration, while ulcerative lesions displayed fewer parasites and organised dermal granulomas with prominent infiltration of lymphocytes and epithelioid cells as previously described [3].

### Dermal infiltration of FasL and TRAIL expressing cells was more prominent in ulcerative as compared to non-ulcerative CL

FasL was not expressed in healthy skin (Fig. 2B) and FasL was not upregulated in epidermal cells during CL. FasL expressing dermal cells were present in both ulcerative and non-ulcerative leishmaniasis and accumulated close the ulcerated epidermis as shown in Figure 2B. FasL expressing cells were predominately detected in deep dermis of non-ulcerative CL but at significantly lower levels as compared to ulcerative lesions (Fig. 2B and D).

The level of TRAIL expression was scored using an arbitrary scale shown in Supplementary figure S1. Low or moderate TRAIL expression was detected in healthy epidermis. As previously shown [8], TRAIL expression was increased in epidermis of both LCL and DCL as compared to healthy controls with no significant difference in TRAIL expression between DCL and LCL (results not shown). TRAIL expressing cells were also present in dermis of both ulcerative and non-ulcerative *L. aethiopica* induced CL with significantly higher expression in dermal inflammatory areas in ulcerative as compared to non-ulcerative lesions (Fig. 2C and E).

We were not able to phenotype the TRAIL or FasL expressing cells due to the lack of access of cryopreserved skin tissue. The formalin, paraffin embedded skin biopsies used in this study display abundant auto-fluorescence and could not be used for multi-fluorochrome labelling and detection of double positive cells by confocal microscopy. Previously, Mustafa et al reported that macrophages in *L. aethiopica* induced CL express FasL [14]. In ulcerative CL caused by *L. major*, we have previously shown infiltration of FasL expressing T cells and macrophages were present in dermis in cryopreserved skin biopsies [7].

### Variable levels of epidermal apoptosis were detected in lesion biopsies

**Figure 3:**
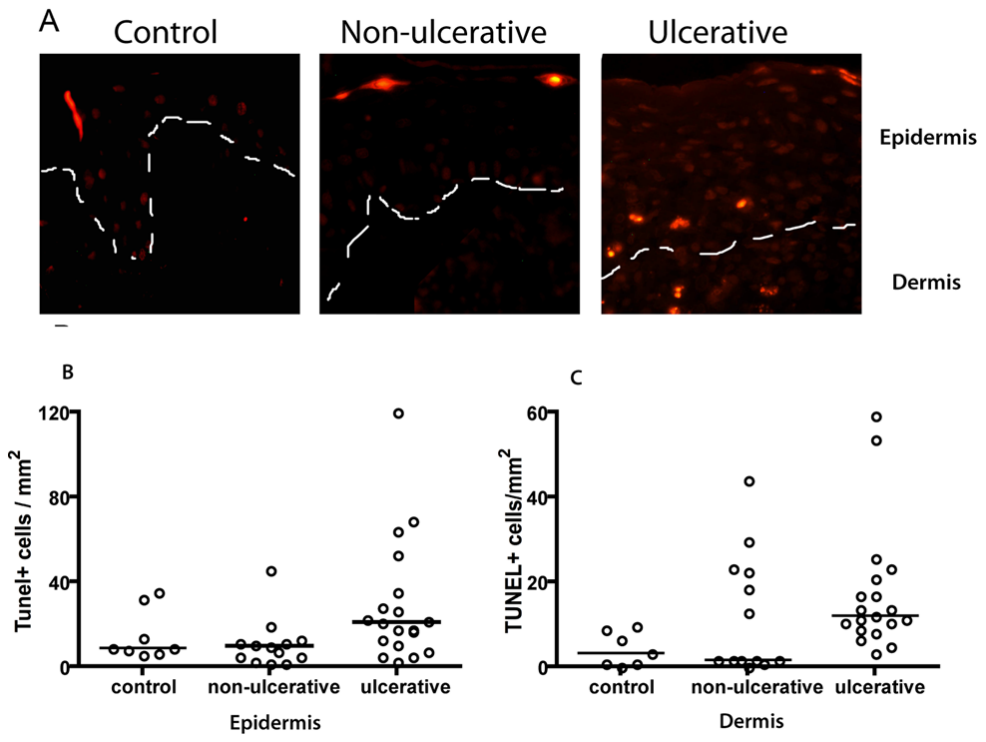
Apoptosis in skin biopsies from ulcerative and non-ulcerative CL. (A) TUNEL staining depicting apoptotic cells (orange) in healthy control (left), non-ulcerative (central) and ulcerative (right) CL. The basal membrane separating epidermis and dermis is marked with a dashed white line. Dot plot showing the number of epidermal (B) and dermal (C) apoptotic cells. The horizontal bar represents the median. No significant differences in number of apoptotic cells were found in the groups investigated.

To determine if the increased expression of TRAIL and FasL correlated to increased keratinocyte apoptosis *ex vivo*, TUNEL staining was performed on biopsies from ulcerative and non-ulcerative leishmaniasis. Previously TUNEL staining on human epidermis showed the same pattern of staining as caspase-cleaved cytokeratin 18, verifying that TUNEL can be used as a marker of apoptosis in *Leishmania* infected skin [7]. The number of epidermal apoptotic cells showed great inter-individual variation in all groups examined (Fig. 3A and B) and ulcerative lesions did not contain significantly higher numbers of epidermal apoptotic cells as compared to non-ulcerative lesions and healthy skin. However, there was a clear trend to a higher number of apoptotic keratinocytes in the ulcerative group. We have previously shown an increase in the number of apoptotic epidermal cells in *L. major* caused ulcerative disease as compared to healthy skin in a cohort of young military recruits with a history of ulcerative leishmaniasis of less than three months upon transfer into a hyperendemic *Leishmania* foci [7]. In the present hospital based study the patient material was collected from a heterogeneous group of patients from endemic areas and the median duration of the disease at the time of tissue collection was longer. Furthermore, infection with *L. major* typically leads to more aggressive tissue destruction as compared to *L. aethiopica* caused infection. Due to the immediate and efficient clearance of apoptotic cells *in vivo* by phagocytic cells such as tissue macrophages, the level of apoptotic cells detected in *ex vivo* biopsies may not reflect the amount of cell death taking place in the tissue. Thus we utilised an *in vitro* experimental set-up in which keratinocytes were exposed to *Leishmania* derived supernatants in the absence of phagocytic cells.

### Induction of keratinocyte apoptosis upon exposure to supernatants from peripheral blood mononuclear cells (PBMCs) stimulated with parasites collected from ulcerative CL

**Figure 4:**
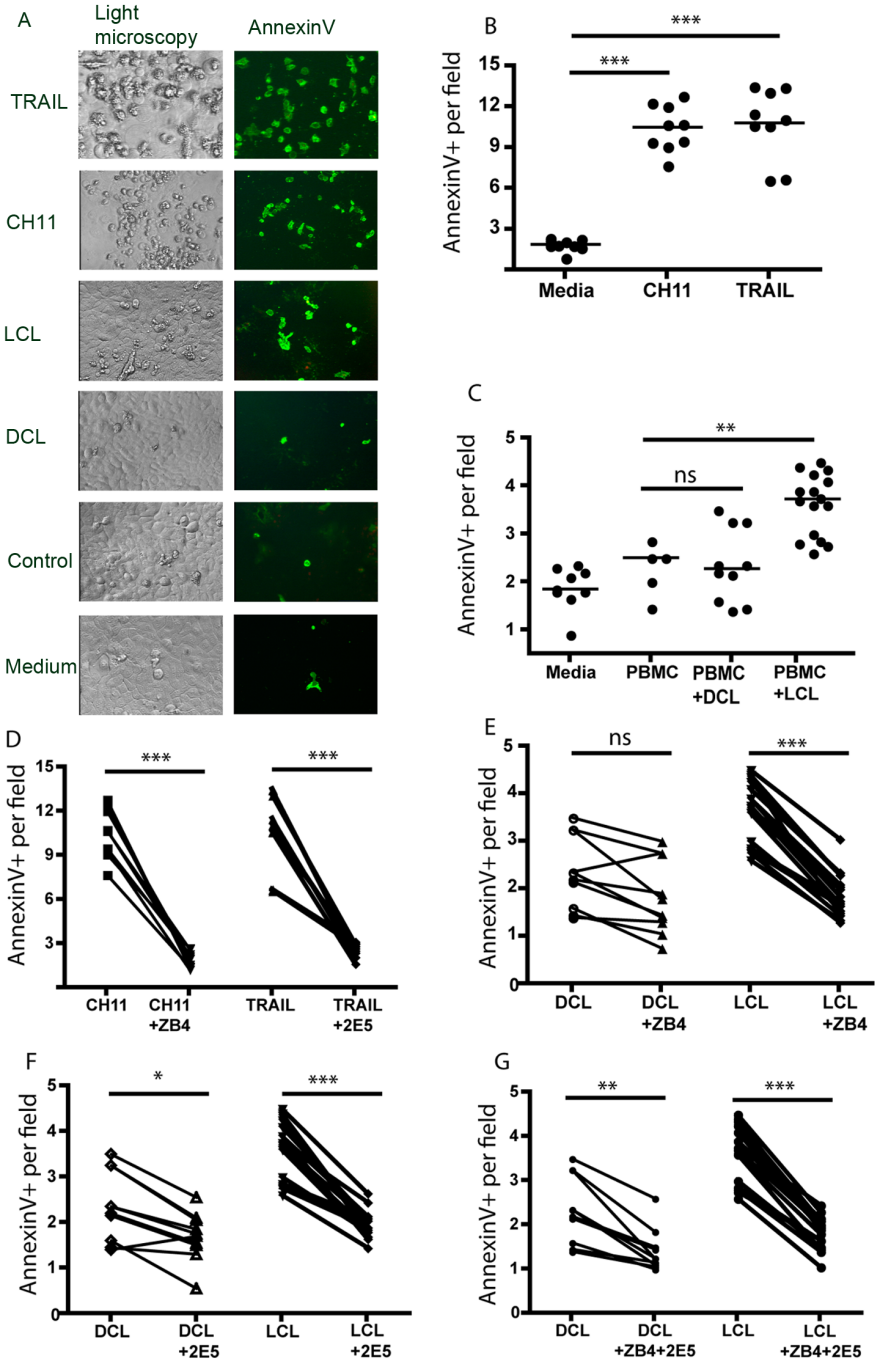
Supernatants from PBMCs stimulated with parasites from LCL patients, but not from DCL patients, induce apoptosis in keratinocytes through FasL and TRAIL signalling. (A) Bright phase photographs (left panel) and AnnexinV (green) plus Propidium Iodide (red) staining (right panel) of monolayers of keratinocytes 20 hrs after addition of anti-Fas (CH11), recombinant TRAIL or supernatants from PBMCs stimulated with promastigotes propagated from ulcerative (LCL) or non-ulcerative (DCL) lesions. CON represents supernatants from unstimulated PBMC and “media alone” keratinocytes in normal media. The pictures depicted did not contain any necrotic cells and thus no Propidium Iodine staining (red) is visible. (B) Dot plot showing induction of keratinocyte apoptosis by addition of anti-Fas (CH11) or recombinant TRAIL. (C) Dot plot showing induction of keratinocyte apoptosis by addition of supernatants from parasite stimulated PBMC cultures as in (A). The horizontal bar represents the median value. (D) Plots showing the inhibitory effect of the Fas blocking antibody ZB4 (1–2 µg/ml) and the TRAIL blocking antibody 2E5 (25 µg/ml) on CH11 (1 µg/ml) and TRAIL (250 ng/ml) induced apoptosis (n = 9). (E–G) Blocking effect of ZB4 and 2E5 on apoptosis induced by DCL (n = 10) and LCL (n = 16) cultures. ***p<0.001, **p<0.01, *p<0.05, ns = not significant.

It has been postulated that distinct subtypes of *L. aethiopica* induce ulcerative and non-ulcerative disease through differential immune-activating properties. To test the apoptosis inducing effect of parasites derived from ulcerative and non-ulcerative lesions, parasites from the different clinical manifestations of CL were obtained from clinical lesions and propagated *in vitro*. Infective promastigotes were used to stimulate PBMC from healthy individuals for seven days and supernatants from such cultures were added to an immortalised keratinocyte cell line sensitive to anti-Fas and TRAIL induced killing (Fig. 4A and B) in which Fas blocking and TRAIL neutralising antibodies completely inhibits apoptosis (Fig. 4D). *Leishmania* promastigotes alone did not induce keratinocyte apoptosis (results not shown) and implicating that the immune activation induced by the parasitic infection was necessary to induce killing of keratinocytes. Supernatants derived from LCL stimulated PBMCs induced significantly more keratinocyte apoptosis as compared to unstimulated PBMC or PBMC stimulated with DCL derived parasites (Fig. 4A and C). Furthermore, keratinocyte apoptosis induced by supernatants from LCL infected PBMC could be inhibited by the addition of Fas blocking antibodies (Fig. 4E) or TRAIL blocking Abs (Fig. 4F). The isotype controls corresponding to TRAIL and Fas blocking antibodies did not have any effect on keratinocyte apoptosis. No synergistic effect was noted when both FasL and TRAIL were inhibited simultaneously (Fig. 4G). The low level of keratinocyte apoptosis induced by supernatants from DCL infected PBMCs could be reduced with TRAIL blocking antibodies but not with Fas blocking antibodies (Fig 4 E–F). TRAIL but not FasL is expressed on HaCaT and the levels of TRAIL increase during exposure to inflammatory supernatants. Possibly TRAIL, but not Fas, blocking antibodies may prevent keratinocyte-keratinocyte killing in the context of mild inflammation.

### Short-term neutralisation of FasL and TRAIL decreased the immunopathology without affecting the infectious burden in a murine model of ulcerative CL

Current treatment alternatives during active CL are aimed at parasite eradication [2] and have little effect on tissue destruction. On the contrary, current treatment regimes result in exacerbation of inflammation leading to increased tissue destruction and scarring. Targeting specific immune mechanisms has proven to be a promising new approach for the therapy of cancer and autoimmune diseases. We were interested to investigate if such approach could be used to decrease the pathology caused by a protozoan infection such as *Leishmania* infection.

The effect of systemic treatment with FasL and TRAIL neutralising antibodies during the ulcerative process during CL was investigated. *L. aethiopica* inoculation in mice does not lead to productive infection or ulcerative disease. Thus *L. major*, causing ulcerative leishmaniasis, was used throughout the *in vivo* experiments. C57BL/6 inoculated with *L. major* developed non-ulcerative lesions followed by self-healing and was thus not a suitable model to follow ulcer development. Addition of sandfly salivary gland homogenate to low dose *L. major* infection in C57BL/6 mice, as previously described [15], did not cause stable and reproducible ulcer development suitable for our purpose and was not pursued beyond pilot experiments. High dose infection in C57BL/6 (Jackson strain) mice bred at Karolinska Institutet caused non-ulcerative lesions followed by necrotic degradation of the ear tissue. Thus, a well-characterised model of ulcerative CL using a low number of metacyclic *L. major* promastigotes injected intradermally into the ear of BALB/c was chosen [16] despite the Th2 bias and strong IL-4 production associated with this model.

**Figure 5:**
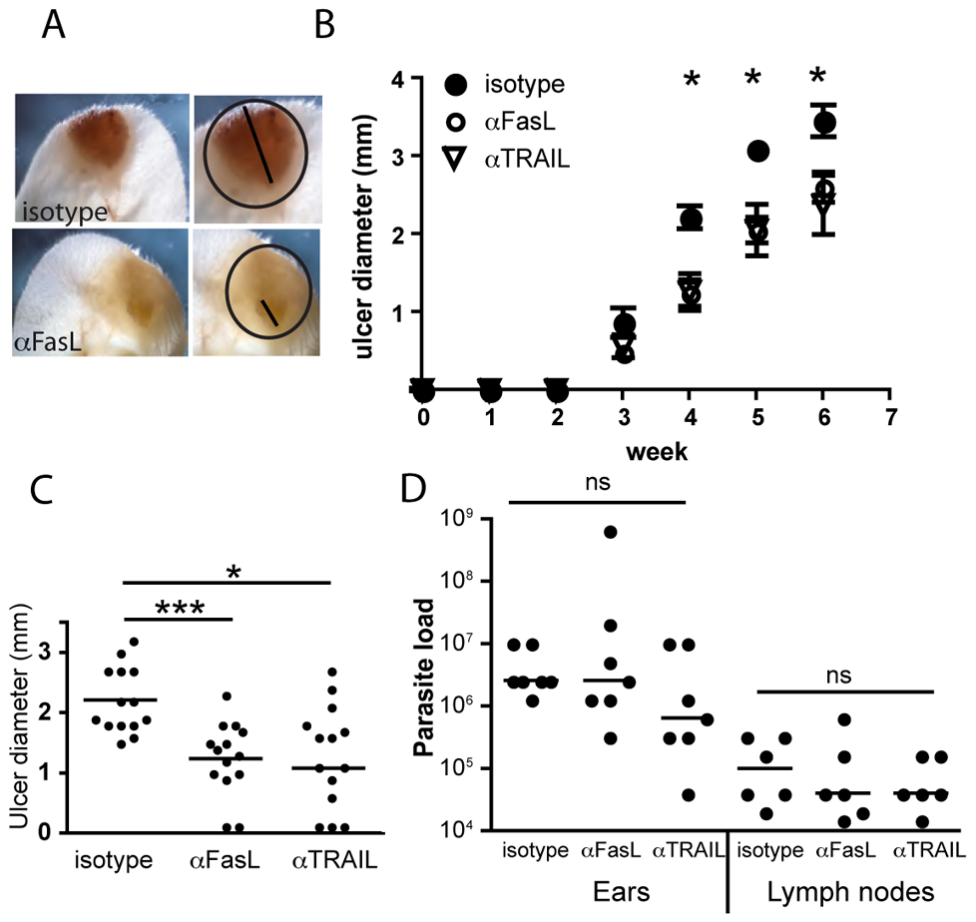
Blocking FasL and TRAIL reduce ulceration during experimental CL. (A) Photograph of *L. major* (strain Friedlin V1) infected ears 4 weeks post infection treated with hamster isotype control (upper row) or anti-FasL neutralising Abs (MFL4, lower row). The right panel depict the diameter of the ulcerated area (line) and the outline of the inflamed, lesional area (circle). (B) Development of ulcer in *L. major* infected BALB/c mice during the treatment with hamster isotype control, anti-FasL (MFL4) or anti-TRAIL (N2B2) was followed over time. The experiment was repeated twice and the pooled results of a total of 14 samples per treatment regime are shown. Mean and standard error of the mean is depicted. (C) Dot-plot diagram showing individual measurements of ulcer size at four weeks after infection. (D) Dot plot diagram showing parasite loads from ears and lymph nodes at four weeks after the infection. Six samples from a representative experiment repeated two times with similar results are depicted. ***p<0.001, *p<0.05, ns = not significant.

Systemic treatment with FasL [12] or TRAIL [13] blocking antibodies was given twice weekly. Hamster and rat isotype control antibodies were given in parallel and there was no difference in ulcer development between the different isotype control antibodies used (results not shown). A clear reduction in the development of ulcers was noted in the treated animals as compared to hamster isotype control treatment (Fig. 5A–C). In spite of reduction of ulceration, neutralisation of FasL or TRAIL was not sufficient to completely inhibit ulcer formation and no synergistic effect was noted by the simultaneous administration of FasL and TRAIL neutralising antibodies.

Insufficient clearance of *L. major* infection has previously been shown in Fas and FasL deficient mice and the treatment strategy used could potentially lead to uncontrolled parasite replication. Impaired control of parasite replication has been shown in Fas and FasL deficient mice [17]–[19] and systemic administration of exogenous recombinant FasL to FasL deficient *(gld)* mice led to elimination of parasites and resolution of cutaneous non-ulcerative lesions [17]. *In vitro* studies have shown that macrophages infected with *L. major* up-regulate their surface Fas expression in response to IFN-γ and as a result become susceptible to CD4+ T cell- induced apoptotic death [17]. No data is available on the evolution of *Leishmania* induced pathology in TRAIL deficient mice. Based on the previous studies in FasL deficient mice, there is a potential risk to exacerbate parasite replication through inhibition of FasL during ulceration. In the model of ulcerative leishmaniasis used in these studies, systemic neutralisation of FasL and TRAIL did not affect increased infectious loads at the primary site of infection (Fig. 5D).

Likewise, the infectious load in the draining lymph node was not altered during treatment, suggesting that dissemination of the infection was not enhanced by short-term neutralisation of Fas/FasL and TRAIL-Rs/TRAIL in this model of CL induced by a low dose of infective parasites.

Using a different strain of *L. major* (LV39) did not lead to ulcer development (Fig. S2 A–C) despite similar parasite loads in ears and draining lymph nodes. Interestingly, the area of inflamed skin surrounding the site of infection was reduced whereas the parasite loads were not affected in mice treated with FasL neutralising antibodies, thus mirroring the results obtained using the ulcerative model induced by *L. major* strain Friedlin V1.

There are several potential explanations to why effect of FasL on parasite loads obtained in this report differs from earlier studies. The data previously published was obtained using a different route of inoculation, a different infectious dose and different genetic backgrounds of the host mice. The Fas (*lpr*) deficient transgenic mice show a profound lymphoproliferative phenotype with half the life expectancy as compared to congenic control mice [20]. Due to alterations in the thymic selection of T cells these mice display a skewed T cell repertoire that in itself may affect the ability to combat parasitic disease independently of peripheral FasL signalling during infection. In contrast, we chose to use short-term inhibition of FasL and TRAIL in immuno-competent mice.

### Antigen specific IFN-γ production is not altered upon FasL neutralisation during ulcerative leishmaniasis in BALB/c mice

IFN-γ production by CD4 cells has been ascribed a critical role in parasite clearance during leishmaniasis through activation of infected macrophages. Although the number of CD4+ T cells, and to a lesser extent the CD8+ T cells, were reduced at the site of infection during FasL neutralisation (Fig. S3A–C), the ratio between CD4:CD8 T cells was identical to control infected mice. The percentage of IFN-γ producing CD4+ T cells at the site of infection was not affected by FasL neutralisation as shown in Figure S3D and further antigen stimulation did not enhance the ex vivo production of IFN-γ (not shown), possibly due to the high amounts of parasitic antigen and antigen presenting cells present in the single cell suspension prepared from ear tissue. In line with the finding that FasL neutralisation did not affect the parasitic load, CD4+ T cell antigen-specific IFN-γ production was not affected by FasL neutralisation as shown in figure S3 E–F.

It has recently been reported that FasL may potentiate the effect of IFN-γ signalling in macrophages, leading to more efficient parasite eradication [21]. This effect was prevented in the presence of IL-4 and we cannot exclude that the lack of effect, on the infectious load during FasL neutralisation was influenced by the high levels of IL-4 production in BALB/c mice. Taken the lack of a reliable ulcerative leishmaniasis model on a different genetic background, this concern could not be addressed in the present study.

### Recruitment of neutrophils into sites of infection is reduced during systemic FasL neutralisation

The mechanisms behind ulceration during cutaneous leishmaniasis are not understood. Necrotic death due to intense inflammation is probably one cause of ulceration during leishmaniasis, but publications on the subject are scarce. In a therapeutic attempt to administer IFN-γ during human CL to enhance parasite killing, side effects in terms of pronounced inflammation was noted [22] and it has been postulated but not properly proven that inflammation leading to tissues destruction is necessary for treatment control. In addition to the pure apoptosis inducing effect of FasL, proinflammatory effects of FasL signalling has been proposed in a number of different settings and in macrophages resistant to FasL mediated killing, FasL signalling leads to TNFα and IL-8 secretion potentially leading to recruitment of neutrophils into sites of infection. Interestingly, neutrophils are recruited into the site of infection during cutaneous leishmaniasis in humans [23], [24] and in mice accumulation of neutrophils have been linked to tissue damage [25]. In the latter study, IL-17 was shown to be the major neutrophil chemoattractant during infection.

**Figure 6:**
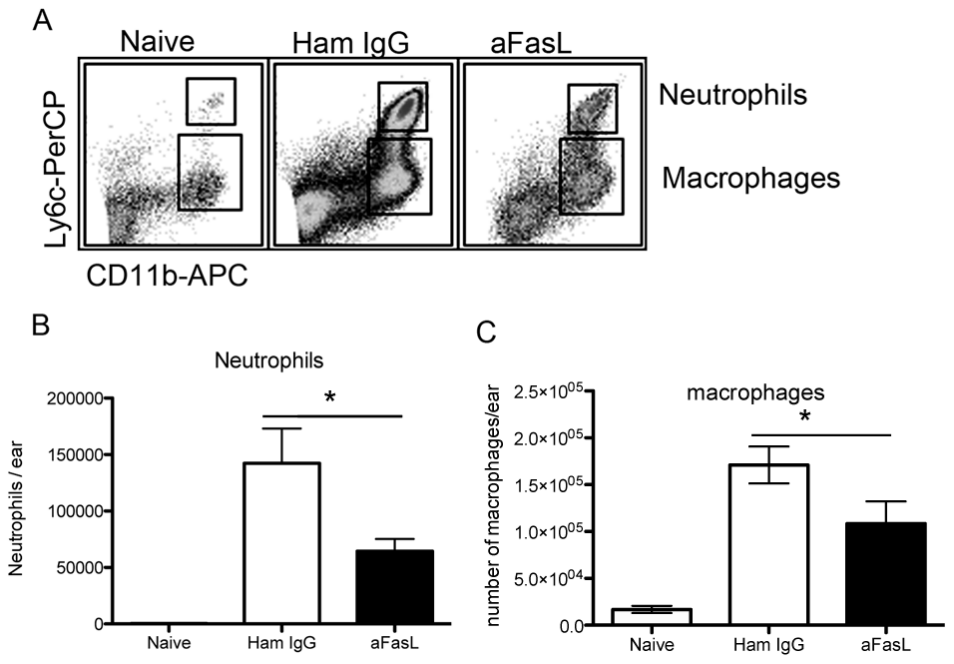
FasL neutralisation lead to reduction of neutrophil migration into *Leishmania* infected skin. (A) Representative FACS plots of single cell suspension gated on live CD45+ cells. Neutrophils were identified as Ly6^high^CD11b^high^ cells. Monocyte/macrophages were identified as Ly6^int/low^ CD11b^high^ cells. (B) Bar graph showing the number of neutrophils in naive and *Leishmania* infected ears. (C) Bar graph showing the number of monocyte/marophages per ear. Eight samples from two separate experiments were pooled four weeks post infection. Mean and SEM are shown.

To test if FasL neutralization leads to an impaired recruitment of neutrophils into the infected skin we enumerated the number of neutrophils and macrophages during FasL neutralisation in parallel to control treated mice. A two-fold reduction in the number of neutrophils was found during FasL neutralisation (Fig 6). Similar results were found in the non-ulcerative model of leishmaniasis obtained by *L. major* (strain LV39) as shown in figure S2. It is possible that a complete block of neutrophils into the site of infection, possibly through targeting IL-17 and FasL simultaneously, would further reduce the ulceration. However, in the context of infection potentially tissue damaging cells (e.g. neutrophils) may be necessary for parasite control. Neutrophils are rapidly recruited to the site of infection after a sand-fly bite and serve as a first host cell to *Leishmania* promastigotes. Neutrophils undergo spontaneous apoptosis within days in peripheral tissues and as *Leishmania* infected, it has been shown neutrophils can facilitate infection [26]. However, in later stages of infection neutrophils probably play a role in controlling the infection through their strong inflammatory, and tissue destructive, function and through activation of *Leishmania* infected macrophages [27]–[29].

In this report, we show that FasL and TRAIL expressing cells infiltrate dermis and that a higher level of expression is present during ulcerative leishmaniasis as compared to non-ulcerative leishmaniasis. *In vitro* experiments confirmed that FasL and TRAIL neutralising antibodies directly inhibit keratinocyte apoptosis in the context of *Leishmania* induced inflammation. The potential role of FasL and TRAIL during *Leishmania* induced ulceration was further strengthened by the reduction of ulceration during systemic neutralisation of both FasL and TRAIL in a murine model of ulcerative leishmaniasis. FasL neutralisation *in vivo* led to a reduction in the recruitment of neutrophils into the site of infection, suggesting additional pro-inflammatory mechanisms of FasL signalling during leishmaniasis.

The results shown here, obtained from human samples and murine *in vivo* experiments, suggest at least two different roles for FasL during skin ulceration in *Leishmania* infection. Firstly, FasL signalling in the inflamed tissue is involved in neutrophil recruitment. Secondly, sFasL induce keratinocyte death. Activated neutrophils are tissue destructive and one can envisage a scenario where neutrophils cause destruction of the basal membrane in areas of intense infection. Through breaking the epidermal-dermal border soluble death ligands get access to keratinocytes leading to direct destruction of the epidermis and ulceration.

Fas-FasL interactions have been implicated in the pathogenesis of drug-induced toxic epidermal necrolysis (TEN), a life-threatening disease characterized by extensive destruction of epidermal keratinocytes [12], [13]. Systemic treatment with intravenous immunoglobulins containing Fas-blocking antibodies limited the ulcerative process during TEN [13] and reduced mortality in several multi-centre analysis. In the case of CL we propose that an adjuvant therapy neutralizing FasL or TRAIL in combination with leishmanicidals could reduce the ulcerative process and subsequent scar formation.

## Supporting Information

Figure S1 Assessment of TRAIL staining in skin biopsies. Representative pictures of TRAIL stainings (DAB, brown) were used to blindly assess the level of TRAIL expression in skin biopsies. Depicted from the left: 1) isotype control 2) healthy skin 3) non-ulcerative leishmaniasis 4–5) ulcerative leishmaniasis.
(1.97 MB TIF)

Figure S2 FasL neutralisation in a non-ulcerative model of CL. (A) Photograph of naive (top row), L. major (strain LV39) infected ears 5 weeks post infection treated with Hamster IgG (mid row) or anti-FasL neutralising antibodies (bottom row). (B) Dot plot diagram showing individual measurements of non-ulcerated lesion size five weeks post infection. (C) Dot plot diagram showing parasite loads from ears and lymph nodes five weeks after infection. Horizontal bars represents median (D) Representative FACS plots of single cell suspension from ear tissue stained for CD45-eFlour450 and Live/Dead YFP. (E) Bar graph showing the number of viable CD45+ cells assessed by FACS analysis of single cell suspension from infected ears. (F) Bar graphs depicting the number of live, CD45+ neutrophils five weeks post infection. *,p<0–05 ** p<0.01. 6–8 samples pooled from two separate experiments are depicted. Mean and standard error of the mean depicted.
(0.49 MB TIF)

Figure S3 T cell infiltration but not IFNγ production are affected by FasL neutralisation. (A) Representative FACS plots of CD4+ and CD8+ T cells gated on live CD45+ TCRβ+ cells from Leishmania infected ears four weeks post infection treated with isotype control (left panel) or antiFasL antibodies (right panel). (B) The number of CD8+ T cells per ear (right panel) and the percentage of CD8+ T cells of total TCRβ+ cells. Four samples from one representative experiments is depicted, in total eight samples were analysed. (C) The number of CD4+ T cells per ear (right panel) and the percentage of CD4+ T cells of total TCRβ+ cells. Four samples from one representative experiments is depicted, in total eight samples were analysed. (D) Representative FACS plots of IFNγ production in live CD45+TCRβ+CD4+ cells four weeks post-infection. (E–F) Representative FACS plots of ex vivo (left panel) and antigen dependent (right panel) IFN γproduction in live CD45+TCRβ+CD4+ cells four weeks post-infection. Representative FACS plots of in total eight samples per group performed in two separate experiments are shown.
(0.25 MB TIF)

## References

[1] Akuffo H, Schurr E, Andersson G, Yamaneberhan T, Britton S (1987). Responsiveness in diffuse versus local cutaneous leishmaniasis is due to parasite differences.. Scand J Immunol.

[2] Lemma A, Foster WA, Gemetchu T, Preston PM, Bryceson A (1969). Studies on leishmaniasis in Ethiopia. I. Preliminary investigations into the epidemiology of cutaneous leishmaniasis in the highlands.. Ann Trop Med Parasitol.

[3] Bryceson AD (1970). Diffuse cutaneous leishmaniasis in Ethiopia. 3. Immunological studies. IV. Pathogenesis of diffuse cutaneous leishmaniasis.. Trans R Soc Trop Med Hyg.

[4] Akuffo HO, Fehniger TE, Britton S (1988). Differential recognition of Leishmania aethiopica antigens by lymphocytes from patients with local and diffuse cutaneous leishmaniasis. Evidence for antigen-induced immune suppression.. J Immunol.

[5] Schurr E, Wunderlich F, Tadesse G (1987). Electron microscopical studies on cutaneous leishmaniasis in Ethiopia. II. Parasite and host cell differences between the localized and the diffuse form.. Acta Trop.

[6] Akuffo H, Maasho K, Blostedt M, Hojeberg B, Britton S (1997). Leishmania aethiopica derived from diffuse leishmaniasis patients preferentially induce mRNA for interleukin-10 while those from localized leishmaniasis patients induce interferon-gamma.. J Infect Dis.

[7] Eidsmo L, Nylen S, Khamesipour A, Hedblad MA, Chiodi F (2005). The contribution of the Fas/FasL apoptotic pathway in ulcer formation during Leishmania major-induced cutaneous Leishmaniasis.. Am J Pathol.

[8] Eidsmo L, Fluur C, Rethi B, Eriksson Ygberg S, Ruffin N (2007). FasL and TRAIL induce epidermal apoptosis and skin ulceration upon exposure to Leishmania major.. Am J Pathol.

[9] Boukamp P, Petrussevska RT, Breitkreutz D, Hornung J, Markham A (1988). Normal keratinization in a spontaneously immortalized aneuploid human keratinocyte cell line.. J Cell Biol.

[10] da Silva R, Sacks DL (1987). Metacyclogenesis is a major determinant of Leishmania promastigote virulence and attenuation.. Infect Immun.

[11] Belkaid Y, Mendez S, Lira R, Kadambi N, Milon G (2000). A natural model of Leishmania major infection reveals a prolonged “silent” phase of parasite amplification in the skin before the onset of lesion formation and immunity.. J Immunol.

[12] Kayagaki N, Yamaguchi N, Nagao F, Matsuo S, Maeda H (1997). Polymorphism of murine Fas ligand that affects the biological activity.. PNAS.

[13] Kayagaki N, Yamaguchi N, Nakayama M, Takeda K, Akiba H (1999). Expression and function of TNF-related apoptosis-inducing ligand on murine activated NK cells.. J Immunol.

[14] Mustafa T, Bjune TG, Jonsson R, Pando RH, Nilsen R (2001). Increased expression of fas ligand in human tuberculosis and leprosy lesions: a potential novel mechanism of immune evasion in mycobacterial infection.. Scand J Immunol.

[15] Belkaid Y, Kamhawi S, Modi G, Valenzuela J, Noben-Trauth N (1998). Development of a natural model of cutaneous leishmaniasis: powerful effects of vector saliva and saliva preexposure on the long-term outcome of Leishmania major infection in the mouse ear dermis.. J Exp Med.

[16] Mendez S, Gurunathan S, Kamhawi S, Belkaid Y, Moga MA (2001). The potency and durability of DNA- and protein-based vaccines against Leishmania major evaluated using low-dose, intradermal challenge.. J Immunol.

[17] Conceicao-Silva F, Hahne M, Schroter M, Louis J, Tschopp J (1998). The resolution of lesions induced by Leishmania major in mice requires a functional Fas (APO-1, CD95) pathway of cytotoxicity.. Eur J Immunol.

[18] Huang FP, Xu D, Esfandiari EO, Sands W, Wei XQ (1998). Mice defective in Fas are highly susceptible to Leishmania major infection despite elevated IL-12 synthesis, strong Th1 responses, and enhanced nitric oxide production.. J Immunol.

[19] Chakour R, Guler R, Bugnon M, Allenbach C, Garcia I (2003). Both the Fas ligand and inducible nitric oxide synthase are needed for control of parasite replication within lesions in mice infected with Leishmania major whereas the contribution of tumor necrosis factor is minimal.. Infect Immun.

[20] Roths JB, Murphy ED, Eicher EM (1984). A new mutation, gld, that produces lymphoproliferation and autoimmunity in C3H/HeJ mice.. J Exp Med.

[21] Chakour R, Allenbach C, Desgranges F, Charmoy M, Mauel J (2009). A new function of the Fas-FasL pathway in macrophage activation.. J Leukoc Biol.

[22] Harms G, Zwingenberger K, Chehade AK, Talhari S, Racz P (1989). Effects of intradermal gamma-interferon in cutaneous leishmaniasis.. Lancet.

[23] Bomfim G, Andrade BB, Santos S, Clarencio J, Barral-Netto M (2007). Cellular analysis of cutaneous leishmaniasis lymphadenopathy: insights into the early phases of human disease.. Am J Trop Med Hyg.

[24] Bretana A, Avila JL, Lizardo G, Convit J, Rondon AJ (1983). Leishmania species: comparative ultrastructure of experimental nodules and diffuse human cutaneous lesions in American leishmaniases.. Exp Parasitol.

[25] Lopez Kostka S, Dinges S, Griewank K, Iwakura Y, Udey MC (2009). IL-17 promotes progression of cutaneous leishmaniasis in susceptible mice.. J Immunol.

[26] Peters NC, Egen JG, Secundino N, Debrabant A, Kimblin N (2008). In vivo imaging reveals an essential role for neutrophils in leishmaniasis transmitted by sand flies.. Science.

[27] Lima GM, Vallochi AL, Silva UR, Bevilacqua EM, Kiffer MM (1998). The role of polymorphonuclear leukocytes in the resistance to cutaneous Leishmaniasis.. Immunol Lett.

[28] McFarlane E, Perez C, Charmoy M, Allenbach C, Carter KC (2008). Neutrophils contribute to development of a protective immune response during onset of infection with Leishmania donovani.. Infect Immun.

[29] Guimaraes-Costa AB, Nascimento MT, Froment GS, Soares RP, Morgado FN (2009). Leishmania amazonensis promastigotes induce and are killed by neutrophil extracellular traps.. Proc Natl Acad Sci U S A.

