# Supplementary figures and images for: Systemic FasL and TRAIL Neutralisation Reduce Leishmaniasis Induced Skin Ulceration
Source: PLoS Negl Trop Dis. 2010 Oct 12;4(10):e844. doi: 10.1371/journal.pntd.0000844 (PMC2953481; doi:10.1371/journal.pntd.0000844)

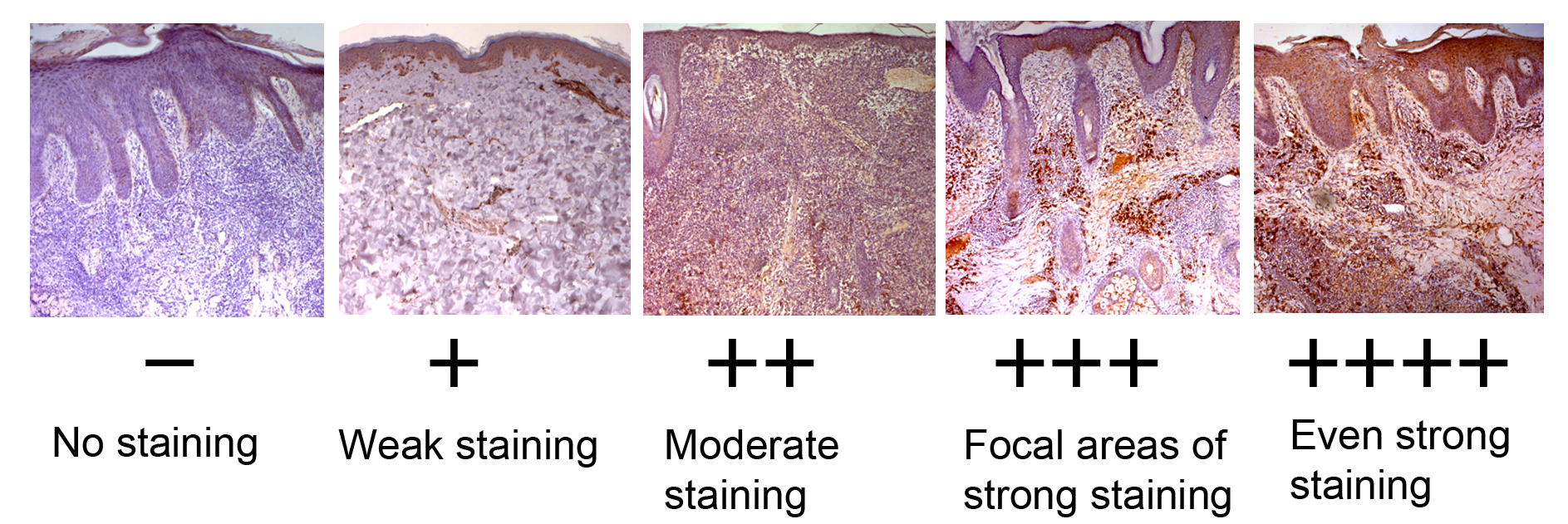

Supplement: Figure S1 — Assessment of TRAIL staining in skin biopsies. Representative pictures of TRAIL stainings (DAB, brown) were used to blindly assess the level of TRAIL expression in skin biopsies. Depicted from the left: 1) isotype control 2) healthy skin 3) non-ulcerative leishmaniasis 4–5) ulcerative leishmaniasis. (1.97 MB TIF) [file pntd.0000844.s001.tif]

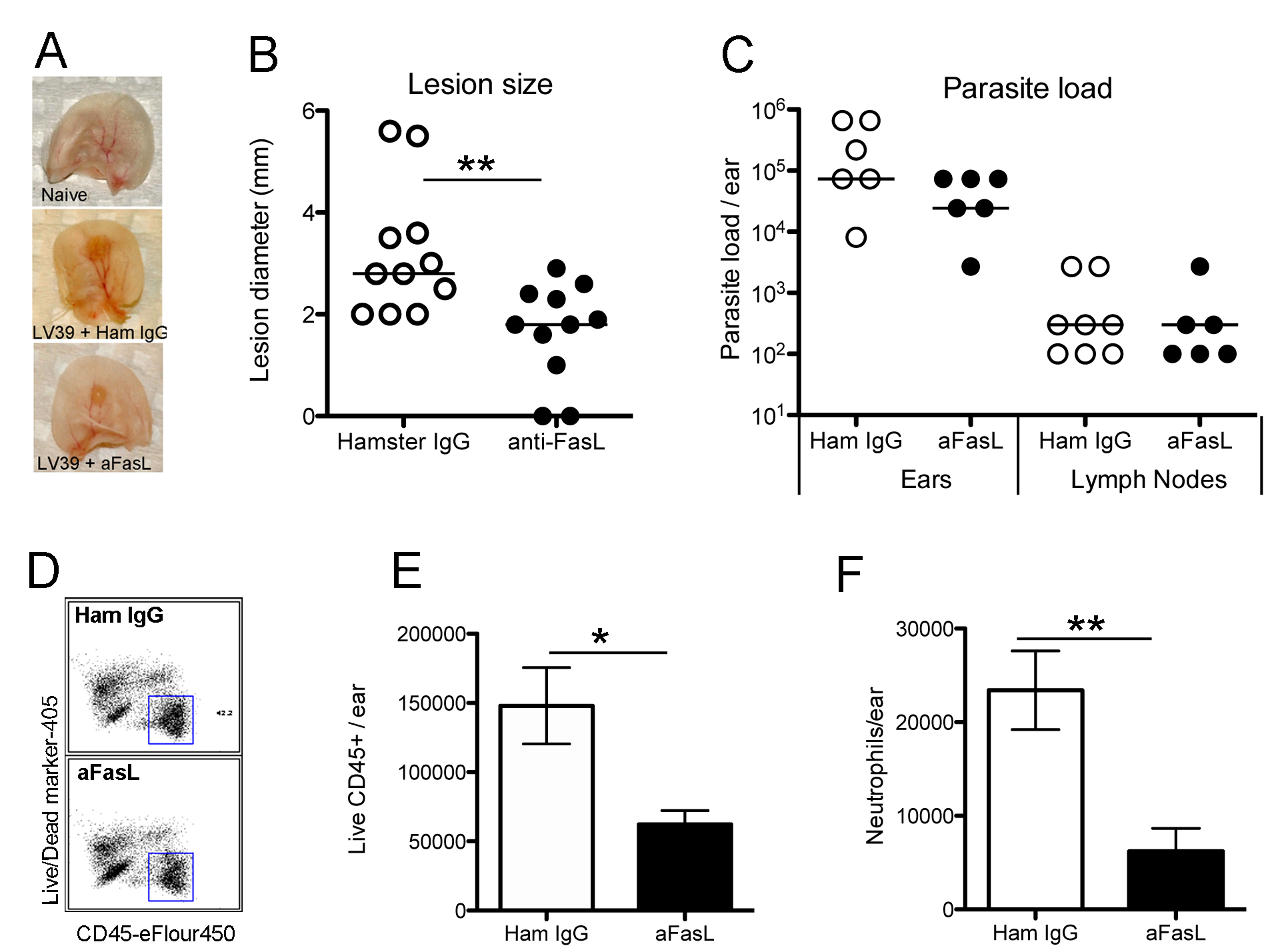

Supplement: Figure S2 — FasL neutralisation in a non-ulcerative model of CL. (A) Photograph of naive (top row), L. major (strain LV39) infected ears 5 weeks post infection treated with Hamster IgG (mid row) or anti-FasL neutralising antibodies (bottom row). (B) Dot plot diagram showing individual measurements of non-ulcerated lesion size five weeks post infection. (C) Dot plot diagram showing parasite loads from ears and lymph nodes five weeks after infection. Horizontal bars represents median (D) Representative FACS plots of single cell suspension from ear tissue stained for CD45-eFlour450 and Live/Dead YFP. (E) Bar graph showing the number of viable CD45+ cells assessed by FACS analysis of single cell suspension from infected ears. (F) Bar graphs depicting the number of live, CD45+ neutrophils five weeks post infection. *,p<0–05 ** p<0.01. 6–8 samples pooled from two separate experiments are depicted. Mean and standard error of the mean depicted. (0.49 MB TIF) [file pntd.0000844.s002.tif]

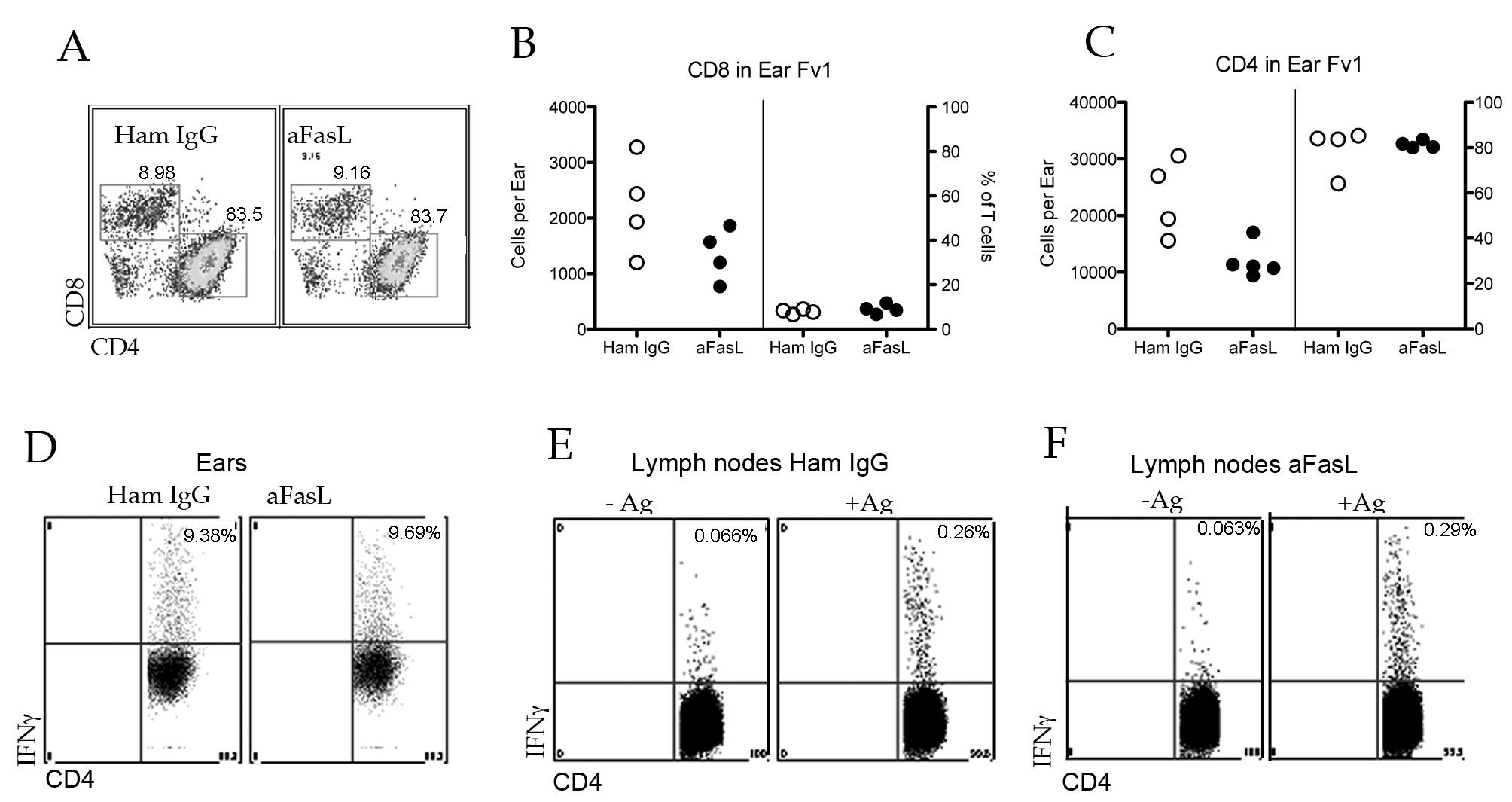

Supplement: Figure S3 — T cell infiltration but not IFNγ production are affected by FasL neutralisation. (A) Representative FACS plots of CD4+ and CD8+ T cells gated on live CD45+ TCRβ+ cells from Leishmania infected ears four weeks post infection treated with isotype control (left panel) or antiFasL antibodies (right panel). (B) The number of CD8+ T cells per ear (right panel) and the percentage of CD8+ T cells of total TCRβ+ cells. Four samples from one representative experiments is depicted, in total eight samples were analysed. (C) The number of CD4+ T cells per ear (right panel) and the percentage of CD4+ T cells of total TCRβ+ cells. Four samples from one representative experiments is depicted, in total eight samples were analysed. (D) Representative FACS plots of IFNγ production in live CD45+TCRβ+CD4+ cells four weeks post-infection. (E–F) Representative FACS plots of ex vivo (left panel) and antigen dependent (right panel) IFN γproduction in live CD45+TCRβ+CD4+ cells four weeks post-infection. Representative FACS plots of in total eight samples per group performed in two separate experiments are shown. (0.25 MB TIF) [file pntd.0000844.s003.tif]
